# *N*-(2,6-Diisopropyl­phen­yl)thio­amide

**DOI:** 10.1107/S1600536812033685

**Published:** 2012-08-01

**Authors:** Bernard Omondi, Demetrius C. Levendis

**Affiliations:** a School of Chemistry, University of KwaZulu-Natal, PO Private Bag X54001, Westville 4000, Durban, South Africa; b Molecular Sciences Institute, School of Chemistry, University of the Witwatersrand, Johannesburg, PO Wits 2050, South Africa

## Abstract

In the crystal structure of the title compound, C_13_H_19_NS {systematic name: *N*-[2,6-bis­(propan-2-yl)phen­yl]carbothio­amide}, mol­ecules assemble *via* N—H⋯S=C hydrogen bonds into helical chains along the *b* axis. The thio­amide moiety, with a *syn* disposition of the N- and C-bound H atoms, is twisted out of the plane of the benzene ring to which it is connected, forming a dihedral angle angle of 77.60 (14)°.

## Related literature

For the synthesis of related aryl­thio­amides, see: Fernandes & Reid (2003 ▶). For related thio­amide structures, see: Chitanda *et al.* (2008 ▶); Michta *et al.* (2008 ▶); Omondi *et al.* (2009*a* ▶); Jarchow & Schmalle (1977 ▶). For related *N*-2,6-disubstituted-aryl­formamides, see: Omondi *et al.* (2008 ▶, 2009*b* ▶,*c* ▶). For phase transformations in *N*-2,6-phenyl­formamides and *N*-2,6-dichloro­phenyl­formamide, see: Omondi *et al.* (2005 ▶); Gowda *et al.* (2000 ▶).
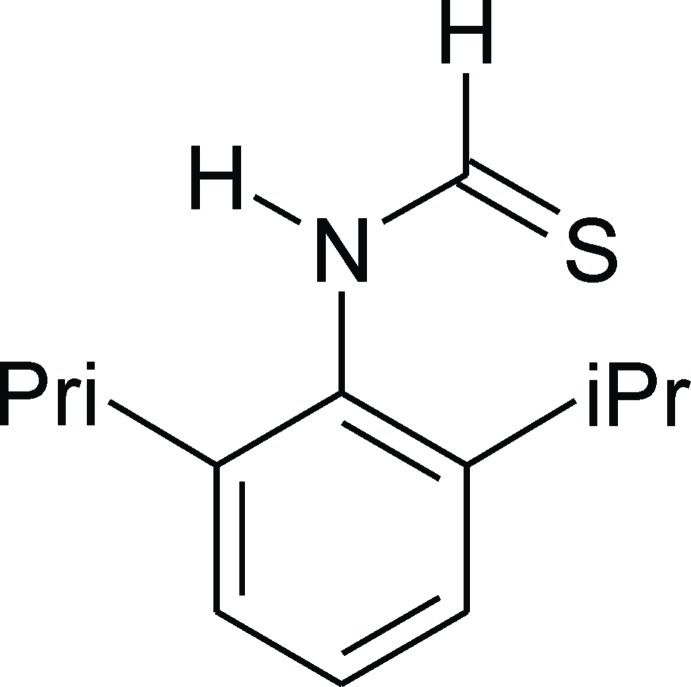


## Experimental

### 

#### Crystal data

C_13_H_19_NS
*M* *_r_* = 221.35
Monoclinic, 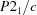

*a* = 9.0230 (12) Å
*b* = 9.3670 (12) Å
*c* = 16.269 (2) Å
β = 101.453 (3)°
*V* = 1347.7 (3) Å^3^
*Z* = 4
Mo *K*α radiation
μ = 0.21 mm^−1^
*T* = 293 K
0.36 × 0.14 × 0.12 mm

#### Data collection

Bruker SMART APEXII CCD area-detector diffractometer
6974 measured reflections
2508 independent reflections
1456 reflections with *I* > 2σ(*I*)
*R*_int_ = 0.098

#### Refinement

*R*[*F* ^2^ > 2σ(*F* ^2^)] = 0.054
*wR*(*F* ^2^) = 0.160
*S* = 0.97
2508 reflections
143 parameters
42 restraints
H atoms treated by a mixture of independent and constrained refinement
Δρ_max_ = 0.23 e Å^−3^
Δρ_min_ = −0.28 e Å^−3^

### 

Data collection: *APEX2* (Bruker, 2005 ▶); cell refinement: *SAINT-Plus* (Bruker, 2005 ▶); data reduction: *SAINT-Plus* and *XPREP* (Bruker, 2005 ▶); program(s) used to solve structure: *SHELXS97* (Sheldrick, 2008 ▶); program(s) used to refine structure: *SHELXL97* (Sheldrick, 2008 ▶); molecular graphics: *PLATON* (Spek, 2009 ▶) and *Mercury* (Macrae *et al.*, 2008 ▶); software used to prepare material for publication: *WinGX* (Farrugia, 1999 ▶) and *PLATON*.

## Supplementary Material

Crystal structure: contains datablock(s) global, I. DOI: 10.1107/S1600536812033685/tk5134sup1.cif

Structure factors: contains datablock(s) I. DOI: 10.1107/S1600536812033685/tk5134Isup2.hkl

Supplementary material file. DOI: 10.1107/S1600536812033685/tk5134Isup3.cml

Additional supplementary materials:  crystallographic information; 3D view; checkCIF report

## Figures and Tables

**Table 1:** Hydrogen-bond geometry (Å, °)

*D*—H⋯*A*	*D*—H	H⋯*A*	*D*⋯*A*	*D*—H⋯*A*
N1—H1⋯S1^i^	0.84 (3)	2.49 (3)	3.316 (2)	166 (2)

Symmetry code: (i) 
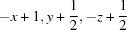
.

**Fig. 1.**
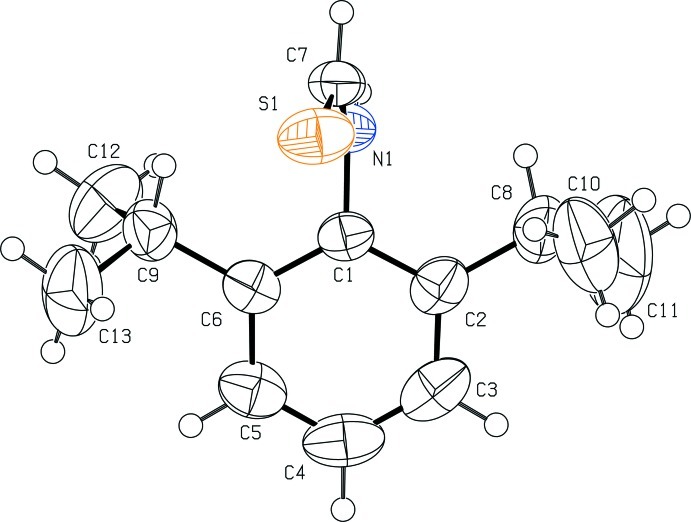
The molecular structure of the title compound. Displacement ellipsoids are shown at the 30% probability level.

**Fig. 2.**
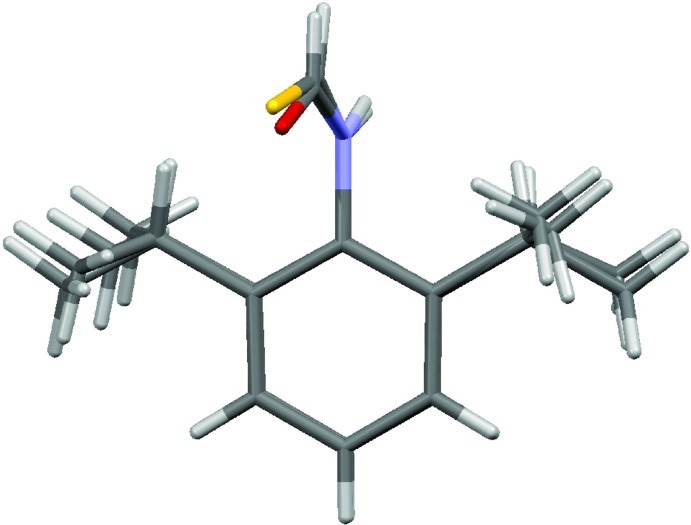
An overlay diagram between structures of the title compound and 2,6-diisopropyl-N-phenylformamide (2).

**Fig. 3.**
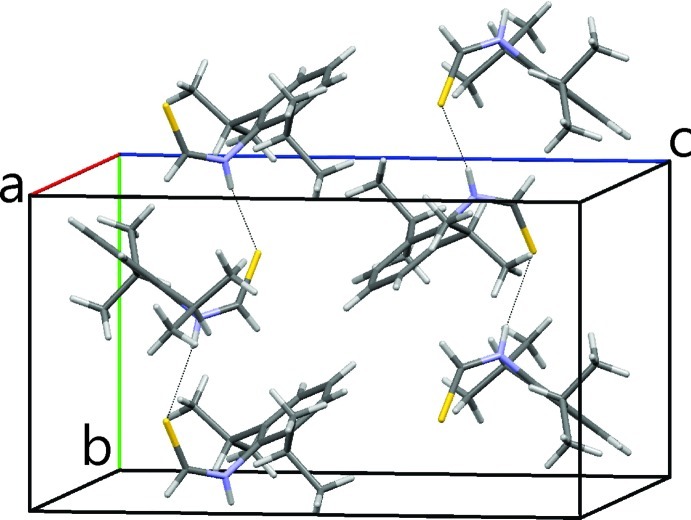
A view down the a axis of the unit cell of the title compound showing the N—H···S═C hydrogen-bonded chains.

## supplementary crystallographic information

### Comment

The synthesis of arylthioamides related to the title compound has been described (Fernandes & Reid, 2003) as have the structures of related thioamides (Chitanda *et al.*, 2008; Michta *et al.*, 2008; Omondi *et al.*, 2009*a*; Jarchow & Schmalle, 1977) and *N*-2,6-disubstituted-arylformamides (Omondi *et al.*, 2009*b*; Omondi *et al.*, 2009*c*; Omondi *et al.*, 2008). In a previous study of 2,6-disubstituted *N*-arylformamides (Omondi *et al.*, 2005), we analyzed the effect of chloro-methyl exchange and the role of weak interactions on the structural and thermal properties of the compounds studied. Phase transitions were observed when the substituents were either both chloride (2,6-dichloro-phenylformamide) or one chloride and one methyl group (2-chloro-6-methyl-phenylformamide); see also Gowda *et al.* (2000). In a subsequent study we analysed the crystal structures of several *N*-arylthioamides (Omondi *et al.*, 2009*a*) with a view to understanding the influence of hydrogen bonds and other weak intermolecular interactions on the conformation and the overall crystal packing of these compounds. The structure of 2,6-diisopropyl-*N*-phenylformamide, 2, was reported recently (Chitanda *et al.*, 2008). In this paper we report on the crystal structure of the analogous 2,6-diisopropyl-*N*-phenylthioamide (1, Fig. 1). Compounds 1 and 2 are isostructural.

The angle between the mean plane through the phenyl ring and the thioamide moiety in 1 is 77.60 (14)°, while in 2 the corresponding angle between the formamide and the phenyl plane is *ca* 79°. The overlay diagram between structures of 1 and 2 is shown in Fig. 2. In 1 chains of molecules are linked *via* N—H···S═C hydrogen bonds. Molecules along these chains are related by screw (2_1_) symmetry as shown by the packing of the molecules in the unit cell (Fig. 3).

### Experimental

The title compound was synthesized using a method similar to one described previously (Omondi *et al.*, 2009*a*). A mixture of the parent formamide and P_2_S_5_ was refluxed in a mixture of THF and benzene for about 60 min (monitoring the reaction progress was by TLC plates). The solvent was then removed *in vacuo* and the product extracted from the remaining solid using benzene. The pale-yellow solution was passed through a column (silica gel) using a 1:1 mixture of hexane and ethyl acetate as the carrier solvent. The product was crystallized directly from the carrier solution. Colourless, block-like crystals were obtained.

### Refinement

The N-bound H atom on the amide was placed according to the observed electron density and allowed to refine freely. The remaining H atoms were positioned geometrically and allowed to ride on their respective parent atoms, with C—H bond lengths of 0.93–0.98 Å and with *U*_iso_(H) = 1.2–1.5*U*_eq_(C). Isopropyl atoms C8–C13 were reported by *PLATON* to have slightly distorted anisotropic displacement parameters (ADP). As a consequence, DELU and SIMU were used in the final refinement to restrain their ADPs to more reasonable values.

### Crystal data

**Table:**

C_13_H_19_NS	*F*(000) = 480
*M**_r_* = 221.35	*D*_x_ = 1.091 Mg m^−^^3^
Monoclinic, *P*2_1_/*c*	Mo *K*α radiation, λ = 0.71073 Å
Hall symbol: -P 2ybc	Cell parameters from 883 reflections
*a* = 9.0230 (12) Å	θ = 2.2–23.3°
*b* = 9.3670 (12) Å	µ = 0.21 mm^−^^1^
*c* = 16.269 (2) Å	*T* = 293 K
β = 101.453 (3)°	Block, colourless
*V* = 1347.7 (3) Å^3^	0.36 × 0.14 × 0.12 mm
*Z* = 4	

### Data collection

**Table:**

Bruker SMART APEXII CCD area-detector diffractometer	*R*_int_ = 0.098
Graphite monochromator	θ_max_ = 25.5°, θ_min_ = 2.3°
ω scans	*h* = −9→10
6974 measured reflections	*k* = −11→11
2508 independent reflections	*l* = −19→16
1456 reflections with *I* > 2σ(*I*)	

### Refinement

**Table:**

Refinement on *F*^2^	Primary atom site location: structure-invariant direct methods
Least-squares matrix: full	Secondary atom site location: difference Fourier map
*R*[*F*^2^ > 2σ(*F*^2^)] = 0.054	Hydrogen site location: inferred from neighbouring sites
*wR*(*F*^2^) = 0.160	H atoms treated by a mixture of independent and constrained refinement
*S* = 0.97	*w* = 1/[σ^2^(*F*_o_^2^) + (0.085*P*)^2^] where *P* = (*F*_o_^2^ + 2*F*_c_^2^)/3
2508 reflections	(Δ/σ)_max_ = 0.002
143 parameters	Δρ_max_ = 0.23 e Å^−^^3^
42 restraints	Δρ_min_ = −0.28 e Å^−^^3^

### Special details

**Table:**

Geometry. All e.s.d.'s (except the e.s.d. in the dihedral angle between two l.s. planes) are estimated using the full covariance matrix. The cell e.s.d.'s are taken into account individually in the estimation of e.s.d.'s in distances, angles and torsion angles; correlations between e.s.d.'s in cell parameters are only used when they are defined by crystal symmetry. An approximate (isotropic) treatment of cell e.s.d.'s is used for estimating e.s.d.'s involving l.s. planes.
Refinement. Refinement of *F*^2^ against ALL reflections. The weighted *R*-factor *wR* and goodness of fit *S* are based on *F*^2^, conventional *R*-factors *R* are based on *F*, with *F* set to zero for negative *F*^2^. The threshold expression of *F*^2^ > σ(*F*^2^) is used only for calculating *R*-factors(gt) *etc*. and is not relevant to the choice of reflections for refinement. *R*-factors based on *F*^2^ are statistically about twice as large as those based on *F*, and *R*- factors based on ALL data will be even larger.

### Fractional atomic coordinates and isotropic or equivalent isotropic displacement parameters (Å^2^)

**Table:**

	*x*	*y*	*z*	*U*_iso_*/*U*_eq_	
C1	0.4519 (3)	0.8487 (2)	0.32841 (15)	0.0460 (6)	
C2	0.2981 (3)	0.8173 (2)	0.31060 (17)	0.0570 (7)	
C3	0.2441 (3)	0.7287 (3)	0.3670 (2)	0.0702 (9)	
H3	0.1419	0.7056	0.3572	0.084*	
C4	0.3383 (4)	0.6750 (3)	0.4364 (2)	0.0733 (9)	
H4	0.2995	0.6166	0.4732	0.088*	
C5	0.4902 (4)	0.7070 (3)	0.45191 (17)	0.0639 (7)	
H5	0.5535	0.6683	0.4987	0.077*	
C6	0.5497 (3)	0.7954 (2)	0.39911 (15)	0.0500 (6)	
C7	0.5833 (3)	0.9161 (3)	0.21651 (16)	0.0571 (7)	
H7	0.6092	0.9935	0.1864	0.068*	
C8	0.1944 (3)	0.8753 (3)	0.2333 (2)	0.0851 (10)	
H8	0.245	0.9589	0.2153	0.102*	
C9	0.7154 (3)	0.8385 (3)	0.42054 (16)	0.0612 (7)	
H9	0.7428	0.877	0.3696	0.073*	
C10	0.1760 (6)	0.7693 (4)	0.1626 (3)	0.1243 (15)	
H10A	0.2739	0.7378	0.1553	0.186*	
H10B	0.1238	0.8135	0.1118	0.186*	
H10C	0.1188	0.6889	0.1755	0.186*	
C11	0.0449 (5)	0.9259 (5)	0.2488 (4)	0.168 (2)	
H11A	−0.0073	0.8477	0.2685	0.252*	
H11B	−0.0146	0.9622	0.1975	0.252*	
H11C	0.0609	1.0001	0.2903	0.252*	
C12	0.7377 (4)	0.9566 (3)	0.4862 (2)	0.0986 (11)	
H12A	0.6709	1.0345	0.4666	0.148*	
H12B	0.8405	0.9894	0.4956	0.148*	
H12C	0.7159	0.9204	0.5377	0.148*	
C13	0.8196 (4)	0.7135 (4)	0.4498 (3)	0.1098 (13)	
H13A	0.8007	0.6786	0.5023	0.165*	
H13B	0.9229	0.7441	0.457	0.165*	
H13C	0.8011	0.6387	0.4087	0.165*	
S1	0.63900 (10)	0.75914 (7)	0.18895 (5)	0.0724 (3)	
N1	0.5084 (2)	0.9469 (2)	0.27423 (14)	0.0530 (6)	
H1	0.485 (3)	1.032 (3)	0.2817 (16)	0.064*	

### Atomic displacement parameters (Å^2^)

**Table:**

	*U*^11^	*U*^22^	*U*^33^	*U*^12^	*U*^13^	*U*^23^
C1	0.0531 (16)	0.0339 (10)	0.0526 (15)	−0.0004 (10)	0.0145 (12)	−0.0042 (10)
C2	0.0520 (17)	0.0388 (12)	0.0803 (19)	0.0005 (11)	0.0134 (14)	−0.0093 (12)
C3	0.0604 (19)	0.0535 (15)	0.104 (3)	−0.0078 (13)	0.0352 (19)	−0.0198 (16)
C4	0.104 (3)	0.0558 (15)	0.073 (2)	−0.0112 (16)	0.049 (2)	−0.0065 (15)
C5	0.088 (2)	0.0606 (14)	0.0467 (15)	0.0014 (15)	0.0216 (15)	−0.0021 (12)
C6	0.0627 (17)	0.0431 (11)	0.0450 (14)	0.0023 (11)	0.0125 (13)	−0.0047 (11)
C7	0.0667 (18)	0.0506 (13)	0.0508 (15)	−0.0163 (12)	0.0040 (14)	0.0105 (12)
C8	0.0568 (19)	0.0644 (16)	0.122 (3)	−0.0023 (14)	−0.0107 (19)	0.0111 (18)
C9	0.0608 (18)	0.0717 (16)	0.0479 (15)	−0.0006 (13)	0.0030 (13)	−0.0004 (13)
C10	0.122 (4)	0.141 (3)	0.090 (3)	0.020 (3)	−0.028 (2)	−0.002 (2)
C11	0.088 (3)	0.166 (4)	0.232 (6)	0.066 (3)	−0.010 (3)	−0.018 (4)
C12	0.094 (3)	0.092 (2)	0.106 (3)	−0.0292 (19)	0.013 (2)	−0.032 (2)
C13	0.072 (2)	0.109 (2)	0.135 (3)	0.0240 (19)	−0.011 (2)	−0.007 (2)
S1	0.0962 (7)	0.0621 (4)	0.0675 (5)	−0.0117 (4)	0.0370 (4)	−0.0061 (3)
N1	0.0592 (14)	0.0341 (9)	0.0634 (14)	−0.0014 (9)	0.0067 (12)	0.0048 (10)

### Geometric parameters (Å, º)

**Table:**

C1—C2	1.392 (3)	C8—H8	0.98
C1—C6	1.395 (3)	C9—C13	1.518 (4)
C1—N1	1.436 (3)	C9—C12	1.523 (4)
C2—C3	1.395 (4)	C9—H9	0.98
C2—C8	1.512 (4)	C10—H10A	0.96
C3—C4	1.367 (4)	C10—H10B	0.96
C3—H3	0.93	C10—H10C	0.96
C4—C5	1.377 (4)	C11—H11A	0.96
C4—H4	0.93	C11—H11B	0.96
C5—C6	1.377 (4)	C11—H11C	0.96
C5—H5	0.93	C12—H12A	0.96
C6—C9	1.521 (4)	C12—H12B	0.96
C7—N1	1.294 (3)	C12—H12C	0.96
C7—S1	1.645 (3)	C13—H13A	0.96
C7—H7	0.93	C13—H13B	0.96
C8—C11	1.497 (5)	C13—H13C	0.96
C8—C10	1.503 (5)	N1—H1	0.84 (3)
			
C2—C1—C6	122.5 (2)	C13—C9—H9	107.8
C2—C1—N1	117.9 (2)	C6—C9—H9	107.8
C6—C1—N1	119.5 (2)	C12—C9—H9	107.8
C1—C2—C3	116.9 (2)	C8—C10—H10A	109.5
C1—C2—C8	121.6 (3)	C8—C10—H10B	109.5
C3—C2—C8	121.5 (3)	H10A—C10—H10B	109.5
C4—C3—C2	121.4 (3)	C8—C10—H10C	109.5
C4—C3—H3	119.3	H10A—C10—H10C	109.5
C2—C3—H3	119.3	H10B—C10—H10C	109.5
C3—C4—C5	120.3 (3)	C8—C11—H11A	109.5
C3—C4—H4	119.8	C8—C11—H11B	109.5
C5—C4—H4	119.8	H11A—C11—H11B	109.5
C6—C5—C4	120.9 (3)	C8—C11—H11C	109.5
C6—C5—H5	119.5	H11A—C11—H11C	109.5
C4—C5—H5	119.5	H11B—C11—H11C	109.5
C5—C6—C1	117.9 (2)	C9—C12—H12A	109.5
C5—C6—C9	120.3 (2)	C9—C12—H12B	109.5
C1—C6—C9	121.7 (2)	H12A—C12—H12B	109.5
N1—C7—S1	128.99 (19)	C9—C12—H12C	109.5
N1—C7—H7	115.5	H12A—C12—H12C	109.5
S1—C7—H7	115.5	H12B—C12—H12C	109.5
C11—C8—C10	111.8 (3)	C9—C13—H13A	109.5
C11—C8—C2	113.8 (4)	C9—C13—H13B	109.5
C10—C8—C2	110.8 (2)	H13A—C13—H13B	109.5
C11—C8—H8	106.7	C9—C13—H13C	109.5
C10—C8—H8	106.7	H13A—C13—H13C	109.5
C2—C8—H8	106.7	H13B—C13—H13C	109.5
C13—C9—C6	112.7 (2)	C7—N1—C1	127.1 (2)
C13—C9—C12	110.7 (3)	C7—N1—H1	119.7 (19)
C6—C9—C12	109.9 (2)	C1—N1—H1	113.2 (19)
			
C6—C1—C2—C3	−0.2 (3)	N1—C1—C6—C9	−0.1 (3)
N1—C1—C2—C3	−176.1 (2)	C1—C2—C8—C11	−138.2 (3)
C6—C1—C2—C8	−179.6 (2)	C3—C2—C8—C11	42.4 (4)
N1—C1—C2—C8	4.5 (3)	C1—C2—C8—C10	94.8 (4)
C1—C2—C3—C4	−0.1 (4)	C3—C2—C8—C10	−84.6 (4)
C8—C2—C3—C4	179.3 (2)	C5—C6—C9—C13	45.2 (4)
C2—C3—C4—C5	−0.4 (4)	C1—C6—C9—C13	−137.9 (3)
C3—C4—C5—C6	1.2 (4)	C5—C6—C9—C12	−78.7 (3)
C4—C5—C6—C1	−1.5 (4)	C1—C6—C9—C12	98.1 (3)
C4—C5—C6—C9	175.5 (2)	S1—C7—N1—C1	−1.3 (4)
C2—C1—C6—C5	1.0 (3)	C2—C1—N1—C7	−103.4 (3)
N1—C1—C6—C5	176.8 (2)	C6—C1—N1—C7	80.6 (3)
C2—C1—C6—C9	−176.0 (2)		

### Hydrogen-bond geometry (Å, º)

**Table:**

*D*—H···*A*	*D*—H	H···*A*	*D*···*A*	*D*—H···*A*
N1—H1···S1^i^	0.84 (3)	2.49 (3)	3.316 (2)	166 (2)

Symmetry code: (i) −*x*+1, *y*+1/2, −*z*+1/2.
